# PLA Nanoplastics Accumulate but Do Not Cause Acute Toxicity to Marine Rotifers, Brine Shrimps, and Zebrafish Embryos

**DOI:** 10.3390/jox15060196

**Published:** 2025-11-12

**Authors:** Doyinsola Suliat Mustapha, Olga Rodríguez-Díaz, Miren P. Cajaraville, Amaia Orbea

**Affiliations:** CBET+ Research Group, Department Zoology and Animal Cell Biology, Faculty of Science and Technology and Research Centre for Experimental Marine Biology and Biotechnology PiE, University of the Basque Country UPV/EHU, Sarriena z/g, E-48940 Leioa, Spain; dm4003@hw.ac.uk (D.S.M.); olga.rodriguezd@ehu.eus (O.R.-D.); mirenp.cajaraville@ehu.eus (M.P.C.)

**Keywords:** bioplastics, toxicity, *Brachionus plicatilis*, *Artemia salina*, *Danio rerio*, polylactic acid, biomarkers, oxidative stress, neurotoxicity

## Abstract

Conventional plastics are widely utilised across industrial sectors and in consumer products. However, the growing use of plastics has led to plastic pollution, including the formation of nanoplastics (NPs), which are harmful to aquatic organisms. Bioplastics are emerging alternatives. They are renewable and/or biodegradable and are supposed to be more environmentally friendly. However, the toxicity and environmental fate of bioplastics are not yet fully understood. This study evaluated the toxicity and fate of commercially available plain or fluorescent polylactic acid (PLA) NPs (250 nm) on aquatic organisms. Confocal microscopy demonstrated the uptake of fluorescent PLA NPs by the test organisms, marine rotifers (*Brachionus plicatilis*), brine shrimps (*Artemia salina*) and zebrafish (*Danio rerio*) embryos. However, the results of the bioassays indicate that plain PLA NPs did not induce acute toxicity in either of the two zooplankton species and did not cause substantial mortality, malformations, or hatching delays in zebrafish embryos at the tested concentrations (up to 100 mg/L). However, brine shrimp showed a significant decrease in ingestion capability. The biochemical biomarkers, catalase activity induction, as an indicator of oxidative stress, and acetylcholinesterase inhibition, as a marker of neurotoxicity, showed no significant alterations compared to the control of both zooplankton species and that of zebrafish embryos. Overall, the findings suggest a pattern of no acute and low sublethal toxicity for the tested plain PLA NPs in the studied organisms. Nonetheless, further research is imperative to comprehensively assess the environmental fate of bioplastics found in various consumer products, as these may contain harmful chemical additives, as well as the effects of prolonged exposure and their impact on physiological parameters, ensuring informed decisions before their widespread commercialisation and presence in the environment.

## 1. Introduction

Plastics are ubiquitous and extensively employed in industrial sectors and everyday consumer goods due to their adaptability as packaging materials, ease of production, and cost-effectiveness [1]. There has been a rise in plastic production since its discovery, reaching an estimated global production of about 413.8 million metric tons (Mt) in 2023 [2]. Environmental processes such as photodegradation, fragmentation, abrasion, and biological degradation contribute to the deterioration of plastic in the environment, leading to the formation of nanoplastics (1–1000 nm; NPs), microplastics (1–1000 μm; MPs), mesoplastics (1–10 mm) and macroplastics (>1 cm) according to the classification proposed by Hartmann et al. [3]. These plastic wastes accumulate and persist in the environment and consequently create favourable conditions for the growth of diverse microbial communities known as the “plastisphere” [4,5]. Plastics can also act as a carrier, facilitating the transport of invasive species [6]. In addition, there is a potential risk of leaching chemical additives and sorbed environmental contaminants (metals, polycyclic aromatic hydrocarbons and other persistent organic compounds) aside from the physical harm and inflammatory effects caused to living organisms upon ingestion, and the potential for bioaccumulation throughout the food chain [7,8].

Several reports have highlighted the toxicity of NPs to aquatic organisms. This toxicity is thought to stem from their extremely small size and the nature of surface functional groups [9,10]. Aquatic organisms, including low-trophic species like plankton, can mistake NPs for food, leading to their ingestion, accumulation and transport through the food chain as they move from lower to higher trophic levels. Other routes of NP exposure are dermal absorption [11] and maternal transfer [12]. The accumulation of NPs in cells, tissues, and organs of aquatic organisms has been reported to disrupt their metabolism, growth, behaviour, and reproduction [13,14,15]. NPs can cause several other effects, including the generation of reactive oxygen species (ROS), the activation of antioxidant enzyme activities, and the disruption of gene expression. These effects have the potential to result in neurotoxicity, genotoxicity, cytotoxicity, and reproductive toxicity, among others [16,17,18,19]. Additionally, NPs can traverse the intestinal barrier and undergo translocation within the intestinal epithelial cells of aquatic vertebrates, as reported by Vagner et al. [20]. They can also breach the highly selective blood–brain barrier, a characteristic not shared by larger plastic particles and induce oxidative stress in the brain, causing deficits in memory and learning ability as reported in zebrafish by Zhou et al. [21].

The production of conventional plastics from petrochemical sources significantly contributes to environmental pollution, leading to biodiversity loss, habitat destruction, greenhouse gas emissions, and exacerbation of global warming. In response to the environmental concerns arising from petrochemical-based plastics, adopting sustainable practices for managing plastic waste has become essential. A promising solution is bioplastics, which are now gaining widespread acceptance across various sectors, including packaging, food and agriculture, engineering, cosmetics, and various biomedical applications. Bioplastic refers to polymers categorised as bio-based, biodegradable, or both [22]. Bio-based plastics are either partially or entirely derived from biomass or natural sources, such as starch, vegetable oils, and organic waste [23,24]. However, despite this promising outlook, the possibility of bioplastics entering the environment through pathways similar to conventional plastics is high. This is largely driven by the rapid expansion of global bioplastic production which currently represent only about 0.5% of total annual plastic production (over 414 million tonnes) but global production capacity is projected to increase substantially from approximately 2.47 million tonnes in 2024 to around 5.73 million tonnes by 2029 [25] and the limited availability of appropriate disposal and recycling options accessible to consumers. Moreover, further research is needed to fully understand the environmental, economic, and social implications of bioplastics across their life cycle [26]. Consequently, there is a pressing need for studies investigating the potential toxicity of bioplastics to aquatic organisms to better evaluate the toxicological risk they might pose to wildlife.

Polylactic acid (PLA) is an aliphatic polyester made mostly of high-purity monomer lactide and derived sources such as sugar and starch. PLA is a biodegradable polymer that possesses excellent biocompatibility, high compostability, processability, thermoplasticity, and antimicrobial activity when combined with various additives [1,27]. Additionally, it has a relatively low production cost compared to other bio-based plastics, providing a competitive edge over conventional plastics [24]. PLA is utilised across diverse areas, including but not limited to packaging, textile, automotive, construction, electronics, cosmetics, and biomedical applications [24,28,29]. The extensive adoption of PLA in various industries reflects the perception that it is the most environmentally friendly choice in polymer production. However, PLA has some drawbacks, which include low crystallinity, inadequate gas barrier, and limited flexibility. To address these limitations, PLA is often blended with other polymers possessing superior gas barrier properties, such as furanoates [30], or combined with poly (hexylene succinate) (PHSu) [31], poly(butylene adipate-co-terephthalate) (PBAT) and poly(propylene adipate) (PPAd) [32]. This blending aims to adjust PLA’s crystallinity, boost degradation rates, and may involve the incorporation of suitable additives like metals and organic and inorganic substances [33,34] for enhanced performance. This blending has sparked safety concerns, particularly for PLA used in food packaging and beverage containers. Some oligomers or additives, termed non-intentionally added substances (NIAS), have been identified with the potential to migrate into food and drinks [35,36] and subsequently enter the environment, posing potential risks to organisms. In this study, we used plain PLA NPs without blending or additives. This approach ensures that any observed biological effects can be attributed directly to PLA itself, rather than to additives or copolymers. It also provides a clearer understanding of the intrinsic impacts of plain PLA NPs on biological systems, against which the additional effects of blended formulations or additive-containing materials can later be compared.

*Brachionus plicatilis* (marine rotifer), *Artemia salina* (brine shrimp), and *Danio rerio* (zebrafish) are commonly used model organisms to assess the impact and toxicity of pollutants. The marine rotifers are adaptable for research due to their small size, high population density, parthenogenetic reproduction, availability of culture techniques, rapid population growth rates, responsiveness to a diverse array of toxic substances, and their significant role in transporting aquatic pollutants across the food web [37,38,39]. *Artemia* sp. (brine shrimp) are small crustaceans recognised as primary consumers in the ocean food web and are the preferred live food source for numerous fish and aquatic invertebrates [40], therefore also playing a crucial role in facilitating the transfer of pollutants throughout the diverse food chains within ocean ecosystems [41,42,43]. Brine shrimp functions as a nonselective filter feeder with a preference for particles smaller than 50 μm, showcasing its potential to absorb various contaminants [42]. Zebrafish is a model freshwater fish. The embryos and larvae are transparent, making them useful for localisation of unlabelled and labelled environmental pollutants, including MPs and NPs [44,45,46]. The larvae’s capability to survive for up to seven days on their yolk sac provides a reliable and cost-effective method for studying the potential adverse effects of environmental pollutants [47].

Addressing the fate of NPs is a challenging issue due to the lack of analytical tools to detect them in exposed organisms and the difficulty of linking potential adverse effects with NP accumulation. In the present study, we took the advantage of the existence of fluorescent and plain PLA NPs of the same characteristics and we combined the use of confocal microscopy for the detection of fluorescent PLA NPs in the investigated organisms with a battery of bioassays and biochemical biomarkers to study the toxicological response in two aquatic invertebrates (rotifer and brine shrimp) and in a model vertebrate species (zebrafish embryos), representing marine and freshwater habitats and different trophic levels. Zooplankton is a primary consumer in the aquatic food chain, serving as energy links to higher trophic organisms, while zebrafish embryos offer insights into potential vertebrate developmental impacts. We hypothesised that PLA NPs would be uptaken by the organisms and could elicit both acute and sublethal responses in the test species.

## 2. Materials and Methods

### 2.1. Obtention and Characterisation of Test Materials

Plain and fluorescent PLA NPs were procured from CD Bioparticles (Shirley, NY, USA). According to the manufacturer’s information, PLA NPs display a density of 1.0 g/cm^3^ and a nominal particle size of 250 nm in diameter. The formulations do not contain any additives. The commercial stock concentration is 1.22 × 10^15^ part/L, equivalent to 10 g/L. The fluorescent particles show an excitation peak at 552 nm and an emission peak at 580 nm. Further characterisation was performed at the General Research Services (SGIker) of the University of the Basque Country. Transmission electron microscopy (TEM) work was performed on a TECNAI G2 20 TWIN (FEI, Eindhoven, Netherlands) operated at 120 kV and equipped with a LaB6 filament. Samples used for TEM analysis were prepared via dispersion in water. A drop of suspension was spread onto a TEM copper grid (300 Mesh) covered by a pure carbon film and dried at ambient temperature. The grid was glow discharge before putting the drop of suspension. The diameter of a random sample of 250 particles of each type was measured on the micrographs (×8700). The behaviour (Z potential and hydrodynamic size) of the plain particles in the exposure media was analysed by Dynamic Light Scattering (DLS, Zetasizer Ultra, Malvern Panalytical, Malvern, UK). For Z potential measurements, DTS1070 cells were used, while for the hydrodynamic size, disposable polystyrene cuvettes were employed. Unless noted otherwise, all other chemicals (reagent grade) used in this study were obtained from Sigma-Aldrich^®^ (now Merck KGaA, Darmstadt, Germany).

### 2.2. Test Organisms

Marine rotifers *Brachionus plicatilis* utilised in acute exposure tests were sourced from a pre-established live culture initially obtained from Power Aquaculture (Mungia, Spain). The rotifers were kept with a constant supply of mild aeration and maintained at an ambient temperature of 22–23 °C, with water salinity set at 27 parts per thousand (ppt) (27 g/L of Tropic Marine^®^ Reef Salt, Wartenburg, Germany, in deionised water). For ingestion tests, rotifers were hatched 24 h before the assay from cysts purchased from Microbiotests^®^ (Gent, Belgium) in 15 ppt water at 28 °C under constant light. Rotifers aged 2–6 h were used for the assay.

Brine shrimps Artemia salina obtained as cysts from Artemia Koral^®^ GmbH (Nürnberg, Germany), were freshly hatched using an Artemia Hatchery (Hobby^®^, Grafschaft, Germany) filled with 30 ppt water. The newly hatched individuals were separated and maintained for an additional 24 h period before being used in acute toxicity and ingestion assays.

Adult zebrafish (AB wild type) stock, sourced from the zebrafish facility at the Parc de Recerca Biomèdica de Barcelona (PRBB), was maintained in a temperature-controlled automated recirculating aquaria system (ZebTec Active Blue, Tecniplast, Italy) at the zebrafish facility of the University of the Basque Country. The water temperature was set at 27 °C, with parameters maintained at 600 µS/cm conductivity and pH 7, under a fixed 12:12 h light-dark photoperiod. Weekly monitoring of nitrogenous compounds in the water was conducted using JBL GmbH kits (Neuhofen, Germany).

### 2.3. Uptake and Localisation of Fluorescent PLA NPs in Zooplankton and Zebrafish Embryos

To assess uptake, organisms were exposed to different concentrations of fluorescent PLA NPs (1, 10, and 100 mg/L) for 48 h or 120 h for zooplankton and zebrafish embryos, respectively, in small Petri dishes. After exposure, individuals were washed five times with their respective control medium and fixed in 4% formaldehyde. Fluorescence imaging was performed using a LSM80 (ZEISS, Oberkochen, Germany) confocal laser scanning microscope, and images were acquired with an illumination range of 561–620 nm. Individuals were mounted in Ibidi glass-bottom Petri dishes. Images of 3–4 individuals per treatment were acquired in both brightfield and fluorescence channels, using z-stack mode (2 µm intervals) to capture the full depth of each specimen. Images were processed using Fiji (ImageJ 1.54f), where orientation was standardised to align individuals, and brightness/contrast were adjusted using non-exposed control organisms as reference, with identical settings subsequently applied to exposed organisms to ensure comparability. Fluorescence quantification was then performed in Fiji by selecting regions of interest (ROIs) with the drawing tools, setting measurements to record “area,” “integrated density,” and “mean grey value,” and extracting values using the Measure function. Background fluorescence was estimated from non-fluorescent regions, and corrected total fluorescence (CTF) was calculated as: CTF = Integrated Density − (Area × Mean background fluorescence).

### 2.4. Acute Toxicity and Ingestion Tests in Zooplankton

Acute toxicity tests followed the guidelines outlined in ISO-19820 [48] for rotifers that were run in 48-well polystyrene (PS) microplates (Sarstedt, Nümbrecht, Germany). Organisms were exposed to concentrations detailed in Table 1. Actively swimming females were carefully chosen using a Nikon SMZ800N (Tokyo, Japan) stereomicroscope and transferred to a Petri dish. Subsequently, 25 individuals were moved to each of the two rinsing wells for each exposure concentration to prevent dilution of concentrations in the testing wells with the rotifer control medium. Finally, five organisms from the rinsing wells were transferred to each of the six treatment replicates containing 0.5 mL of the test solution (n = 30). The microplates were covered and maintained in the dark at 25 ± 1 °C for 24 and 48 h. As the bioassay was of short duration, the organisms were not fed during the test. At 24- and 48-h post-exposure (hpe), the microplate was examined under the stereomicroscope, and the number of dead rotifers in each well was documented. Organisms were considered dead if they exhibited no movement during 10 s of observation. The validity of the test was established if the mortality rate in the negative control did not exceed 10%.

The same procedure was followed for brine shrimps, but in this case, 24-well PS microplates filled with 2 mL of the testing solution were used.

The ingestion test with newly hatched rotifers (2–6 h old) and 24 h post-hatch (hph) brine shrimp nauplii was conducted following the protocol for rotifers established by [49], utilising 24-well microplates with 4 replicates containing 15 individuals per treatment for both rotifers and brine shrimps (n = 60) per concentration. Test individuals were exposed to 750 µL of test media at concentrations indicated in Table 1. The microplate was then incubated in the dark at 25 °C. After 45 min of exposure, the microplates were thoroughly examined to ensure no mortality. Subsequently, 30 µL of 5.8 µm diameter red-dyed microspheres (Polysciences^®^ Inc., Warrington, PA, USA) were added to each well to obtain a concentration of 2.5 × 10^5^ parts/mL. Following a 15-min feeding period, 3–5 drops of 10% formalin were introduced to each well to fix the organisms and facilitate test scoring. Organisms displaying red guts were classified as feeding, while those without were considered non-feeding or fasting (Figure S1). The assessment focused solely on observing the presence or absence of red colour in the gut, rather than quantifying the quantity of microspheres in each animal. To ensure the validity of the test, the ingestion rate in the control group needed to surpass 80%, as specified by Snell [49].

**Table 1:** PLA NP concentrations used in bioassays, expressed in terms of mass and the number of particles per volume. Concentration unit conversion from mg/L to particles/L was made according to Leusch and Ziajahromi [50] based on the nominal nanoparticle size (250 nm) and density (1.0 g/cm^3^). Concentrations used for biomarker analysis are in bold.

Mass Concentration (mg/L)	Number Concentration (Particles/L)
**100**	**1.22 × 10^13^**
10	1.22 × 10^12^
**1**	**1.22 × 10^11^**
0.1	1.22 × 10^10^
**0.01**	**1.22 × 10^9^**

### 2.5. Zebrafish Embryo Toxicity Test and Apoptosis Assay

The assessment of acute effects in zebrafish embryos was based on the OECD TG236 [51] guideline. The day before the test, male-female pairs were confined in individual breeding traps with slot dividers in PS holding tanks (Tecniplast) several hours before dark conditions. On the following day, before light conditions, dividers were removed to facilitate mating. Eggs, collected 20–40 min after spawning, were examined under a SMZ800N stereomicroscope (Nikon, Tokyo, Japan) to ensure proper development. NP stock suspensions were diluted at concentrations indicated in Table 1 with embryo water (0.06 g/L of Instant Ocean© salt, Blacksburg, VA, USA, in deionised water). Selected embryos were placed in small Petri dishes containing the corresponding exposure media before being transferred to 24-well PS microplates filled with 2 mL of the respective exposure media and controls (embryo water), with three replicates per exposure concentration, totalling 30 embryos per treatment.

Analysis of embryo development occurred at 72-, 96-, and 120-h post-fertilisation (hpf). The assessment focused on the following parameters: (1) mortality, where embryos displaying characteristics such as egg coagulation, absence of somite formation, detachment of the yolk sac, and lack of heartbeat, were considered dead; (2) hatching—instances of prolonged hatching events; and (3) malformations, encompassing spinal/tail deformities, pericardial oedema, and yolk sac oedema. Representative brightfield micrographs capturing both abnormalities and normal development were taken using a Multizoom AZ100 Microscope (Nikon, Tokyo, Japan) coupled to the Nikon NIS-Elements F^®^ software (v. 4.30).

At 120 hpf, ten live larvae from the acute toxicity tests were stained with acridine orange for apoptotic cell detection. Two mL of 10 µM acridine orange solution (Invitrogen™, Darmstadt, Germany) were added to previously rinsed wells [52]. After 30 min, the dye was removed, and the larvae were rinsed at least three times before being anaesthetised with benzocaine for observation under the AZ100 fluorescence stereomicroscope (Nikon, Tokyo, Japan). Apoptotic cells, characterised by fluorescent green dots, were promptly observed and microphotographed under both brightfield and the green fluorescent protein (GFP) filter (excitation at 460 nm and emission at 500 nm). Fluorescence quantification was performed as described above.

### 2.6. Biochemical Biomarkers

For the assessment of the biomarker responses, three concentrations of those used in the bioassays were selected (Table 1): the lowest (0.01 mg/L), the intermediate (1 mg/L) and the highest (100 mg/L).

#### 2.6.1. Sample Preparation

In the case of rotifers, 10,000 individuals per replicate were collected from the laboratory culture and incubated in 20 mL of exposure medium in Falcon tubes for 24 h. For brine shrimps, 50 individuals per replicate at 24 hph were exposed for 24 h in 10 mL of the test medium. In both instances, 5 replicates were prepared for each concentration. Following exposure, zooplankton samples were gathered in 1.5 mL microtubes through filtering with 500 µL of homogenisation buffer (0.1 M sodium phosphate buffer, pH 7.4, containing 150 mM KCl and 1 mM ethylene-diaminetetraacetic acid (EDTA)). All samples were promptly frozen and stored at −80 °C until utilised for analysis.

Biomarker responses in zebrafish larvae were evaluated at 120 h of exposure. A minimum of 50 embryos per replicate were placed into Petri dishes filled with 40 mL of exposure medium at the designated concentrations (Table 1). These dishes were left undisturbed until reaching 120 hpf. Subsequently, 40 larvae per treatment concentration were collected after the exposure, anaesthetised using benzocaine solution and rinsed twice with cold 0.1 M sodium phosphate buffer. The larvae were swiftly transferred to 1.5 mL Eppendorf tubes containing 500 μL of sodium phosphate buffer (pH 7.8) and then rapidly frozen and stored at −80 °C for subsequent utilisation in biomarker analysis [53].

#### 2.6.2. Catalase (CAT) Activity

Catalase activity was assessed following the methodology outlined by Aebi [54], with modifications for UV 96-well microplates (Thermo Fisher Scientific^®^, Waltham, MA, USA). For the zooplankton assay, 20.28 mM H_2_O_2_ was used as substrate in 0.1 M sodium phosphate buffer at pH 7.4, while for the zebrafish assay, 50 mM H_2_O_2_ in 0.1 M potassium phosphate buffer at pH 7 was used. A volume of 5 µL of either the sample or standard dilution (ranging from 0 to 20.28 mM or to 50 mM) was added to each well. The plate was read at 240 nm for 4–5 min at intervals of 25 s in a Cytation 5 reader (Agilent, Santa Clara, CA, USA). The results were normalised to protein concentration and expressed in µmol/min/mg protein.

#### 2.6.3. Acetylcholinesterase (AChE) Activity

The assay was conducted following the protocol outlined by Ellman et al. [55], with adjustments made to the measuring time and sample volume (7.5 µL) to meet the requirements of zooplankton samples. The hydrolysis of acetylthiocholine iodide (156 mM) was examined in a reaction medium consisting of 0.1 M sodium phosphate buffer (pH 7.2) and 1.6 mM of 5,5′-dithiobis-(2-nitrobenzoic acid) (DTNB). The 96-well microplate was read for 30 min at 412 nm to monitor the increase in absorbance resulting from the reaction of thiocholine with DTNB, leading to the formation of the yellow-coloured product 5-thio-2-nitrobenzoate (TNB). Enzyme activity was calculated using an extinction coefficient (ɛ) of 14,150 1/M cm. The specific enzyme activity was expressed as nmol of acetylthiocholine iodide hydrolysed in one minute per mg of protein (nmol/min/mg prot).

#### 2.6.4. Protein Concentration

The protein concentration was determined using Lowry’s method in 96-well microplates, employing the organisms’ specific homogenisation buffer and the DC Protein Assay Kit (Bio-Rad^®^, Hercules, CA, USA). The microplate was read at 750 nm after 15 min of adding 5 µL of the standard dilution or sample to each well along with the assay reagents. The calibration curve was constructed using bovine gamma-globulin (Bio-Rad^®^) at concentrations ranging from 0 to 1.5 mg/mL, diluted with the homogenisation buffer. The protein content results from each sample were utilised for normalising acetylcholinesterase and catalase activities.

### 2.7. Statistical Analysis

Calculation of the LC50 and EC50 values, hatching time and biomarker activity were performed with the aid of the SPSS^®^ statistical package (SPSS 27.0, IBM Analytics, Armonk, NY, USA). Binomial logistic regression was calculated with the R 4.3.2 software package as described in Orbea et al. [56] for the bioassay data. Estimation of parameters was performed using the penalised maximum likelihood proposed by Firth [57] whenever convergence was not obtained using the maximum likelihood method [58].

Normality and homogeneity of variance of the fluorescence quantification data were assessed using the Shapiro–Wilk and Levene tests, respectively. Based on the results, the Kruskal–Wallis test, followed by Benjamini–Hochberg correction for multiple comparisons, was applied to apoptosis assay data and PLA NP fluorescence quantification in brine shrimps and zebrafish embryos. One-way ANOVA followed by Tukey’s post hoc test was used for PLA NP fluorescence quantification in rotifers and for biochemical biomarker data.

All statistical analyses were conducted with a significance threshold of *p* < 0.05.

## 3. Results

### 3.1. Secondary Characterisation of PLA NPs

**Figure 1:**
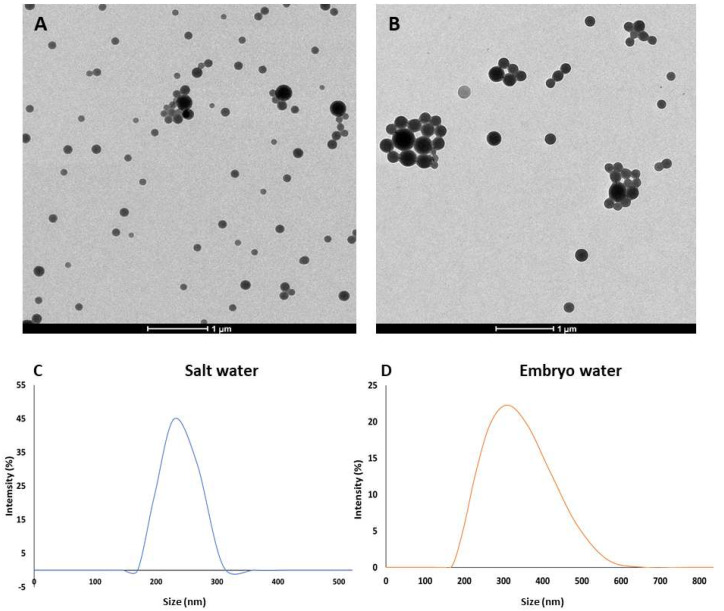
Transmission electron micrographs of plain (**A**) and fluorescent PLA NPs (**B**). Hydrodynamic size of the plain PLA NPs at a concentration of 100 mg/L in salt water (**C**) and in embryo water (**D**).

TEM micrographs of both plain and fluorescent PLA NPs are shown in Figure 1A,B. The NP size measured in a random sample of 250 NPs was 141.35 ± 38.37 nm (min: 82.66 nm; max: 309.33 nm; median: 133.57 nm) for plain PLA NPs and 259.34 ± 158.98 nm (min: 94.66 nm; max: 1127.97 nm; median: 213.33 nm) for fluorescent PLA NPs. Overall, NPs were well dispersed, although some aggregates could be observed, especially in the fluorescent PLA NPs, where larger particles were found. Results of the DLS analysis of plain PLA NPs showed that at a concentration of 100 mg/L, the hydrodynamic particle diameter was 266.94 ± 23.47 and 311.37 ± 27.58 nm in 30 ppt salt water (artificial marine water) and in zebrafish embryo water, respectively (Figure 1C,D). In both cases, particles showed low Z-potential values: 0.88 ± 1.86 mV and 2.35 ± 0.95 mV in salt water and zebrafish embryo water, respectively.

### 3.2. Uptake and Localisation of Fluorescent PLA NPs

**Figure 2:**
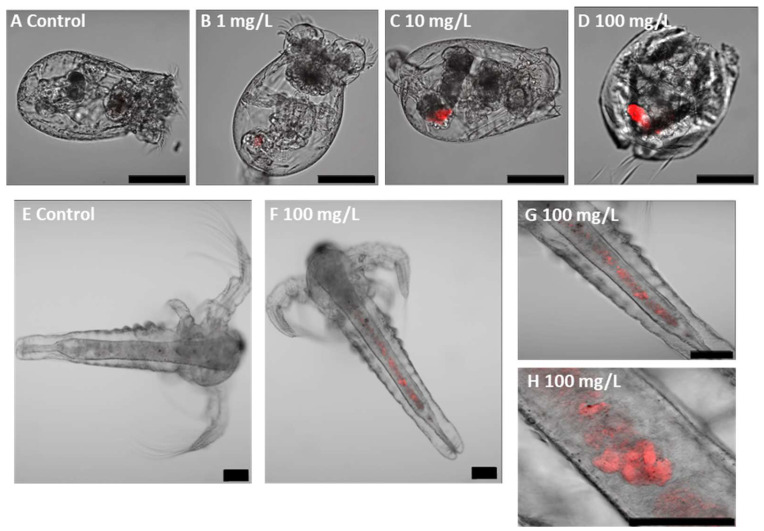
Fluorescent PLA NP uptake and localisation in rotifers (**A**–**D**) and brine shrimps (**E**–**H**) after 48 h of exposure. Scale bars (**A**–**G**): 100 µm. Scale bar (**H**): 50 µm.

**Figure 3:**
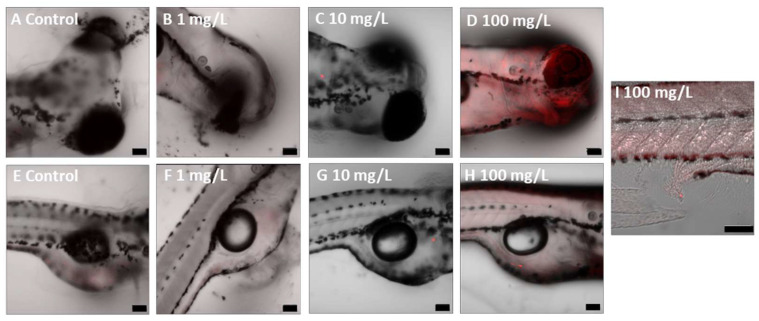
Fluorescent PLA NP uptake and localisation in zebrafish embryos after 120 h of exposure. (**A**–**D**) embryo head area. (**E**–**H**) yolk sac area. (**I**) cloaca. Scale bars: 100 µm.

Confocal microscopy demonstrated the uptake of fluorescent PLA NPs by the exposed organisms and their subsequent localisation within specific body regions, while control individuals did not show fluorescence signals. In zooplankton, fluorescence was primarily observed in the digestive tract (Figure 2) and showed a concentration-dependent increase in intensity, although this trend was not statistically significant (Figure S2). In zebrafish embryos, PLA NPs were detected both in the digestive tract and in the eye region, with additional evidence of excretion through the cloaca (Figure 3). The fluorescent intensity at the highest concentration (100 mg/L) was significantly greater than at the lower concentrations (1 and 10 mg/L).

### 3.3. Acute Toxicity and Ingestion Impairment in Zooplankton

**Figure 4:**
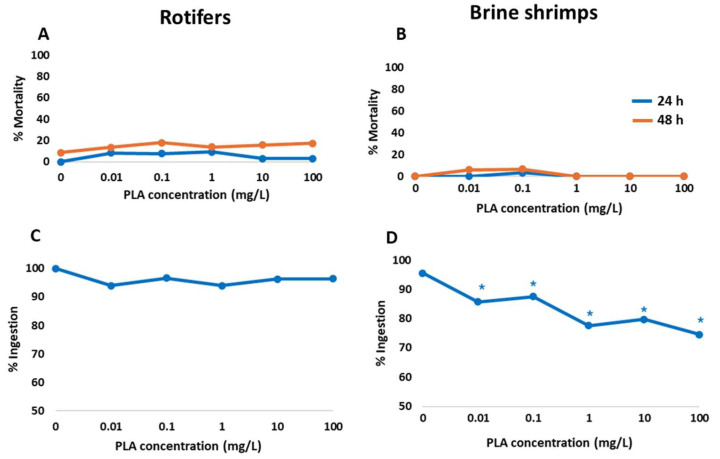
Results of the mortality (**A**,**B**) and ingestion (**C**,**D**) tests in rotifers and brine shrimps exposed to different concentrations of PLA NPs. Asterisks indicate significant differences compared to the control.

Results of the acute toxicity and ingestion assays of zooplankton with PLA NPs are shown in Figure 4. No significant increased mortality was observed in either rotifers or in brine shrimps when compared to the control groups at both 24 h and 48 h of exposure (Figure 4A,B, Table S1). Estimation of the LC50 value was only possible in rotifers for the 48 h exposure duration (LC50 = 763 mg/L), due to very low toxic effects in the rest of the cases, and the value exceeded the highest concentration tested.

Exposure to PLA NPs did not significantly affect rotifer ingestion ability across all tested concentrations (Figure 4C), and accordingly, an EC50 value could not be calculated. Brine shrimps exposed to PLA NPs experienced a significant decrease (Table S2) in their ingestion ability across all tested concentrations, following a dose-dependent pattern. Ingestion was reduced up to 76% when exposed to the highest PLA concentration (Figure 4D). During the test, brine shrimps were observed ingesting and egesting the red microspheres. The estimated EC50 value exceeded the highest concentration tested (EC50 = 253.561 mg/L).

### 3.4. Zebrafish Embryo Toxicity and Cell Death

**Figure 5:**
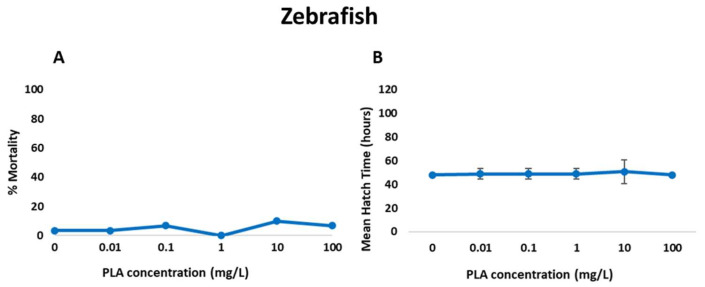
Effect of different concentrations of PLA NPs on zebrafish embryo development: (**A**) mortality rate at 120 h and (**B**) mean and standard deviation of hatching time of zebrafish larvae.

**Figure 6:**
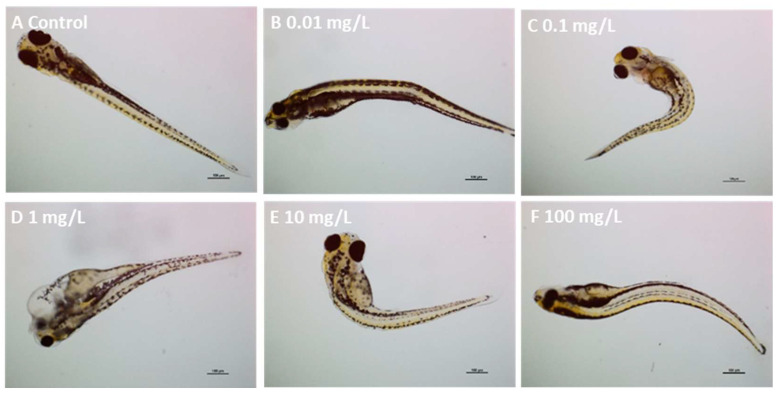
Representative micrographs of zebrafish embryos exposed to PLA NPs for 120 h. (**A**) control embryo showing normal development; (**B**) embryo exposed to 0.01 mg/L PLA NPs showing tail curvature. (**C**) embryo exposed to 0.1 mg/L PLA NPs showing spinal deformity; (**D**) embryo exposed to 1 mg/L PLA NPs showing pericardial and yolk sac oedema; (**E**) embryo exposed to 10 mg/L PLA NPs showing pericardial, yolk sac oedema and spinal deformity; (**F**) embryo exposed to 100 mg/L PLA NPs showing tail curvature. Scale bars: 100 µm.

Results from embryo acute toxicity assays are depicted in Figure 5. PLA NPs did not provoke significant effects on embryo survival (Table S3) or mean hatch time. Accordingly, background levels of malformation, such as spinal deformity, yolk sac and pericardial oedema and curved tail, were observed in all treatments and the control groups (Figure 6, Table 2). Therefore, no increased malformation prevalence was recorded in exposed embryos (Table S4). The estimated LC50 values for mortality and malformation for PLA NPs (732 mg/L and 279 mg/L, respectively) exceeded the highest tested concentration.

**Figure 7:**
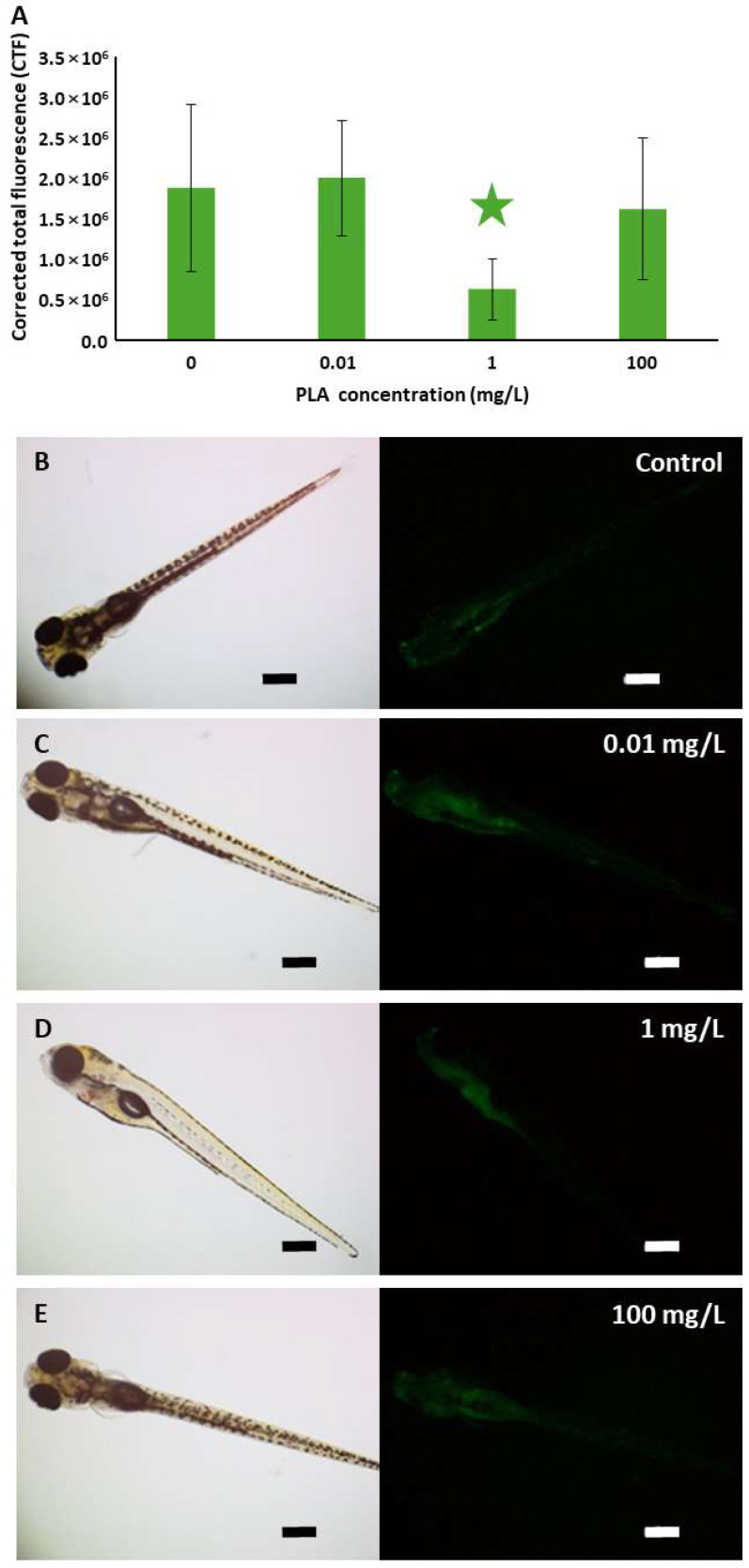
(**A**) Quantification of the fluorescence signal of the acridine orange staining in zebrafish embryos at 120 hpf to detect cell death. The star indicates significant differences between the group exposed to 1 mg/L and the three other experimental groups. (**B**–**E**) Representative brightfield and fluorescence micrographs of zebrafish embryos of the four experimental groups. Scale bars: 100 µm.

Quantification of the fluorescence signal from acridine orange staining indicating the presence of apoptotic cells revealed no significant differences between control and exposed organisms, except for those treated with 1 mg/L, which showed a significantly weaker signal than the others (Figure 7). Thus, exposure to PLA NPs did not increase cell death in zebrafish embryos.

### 3.5. Biomarker Responses

**Figure 8:**
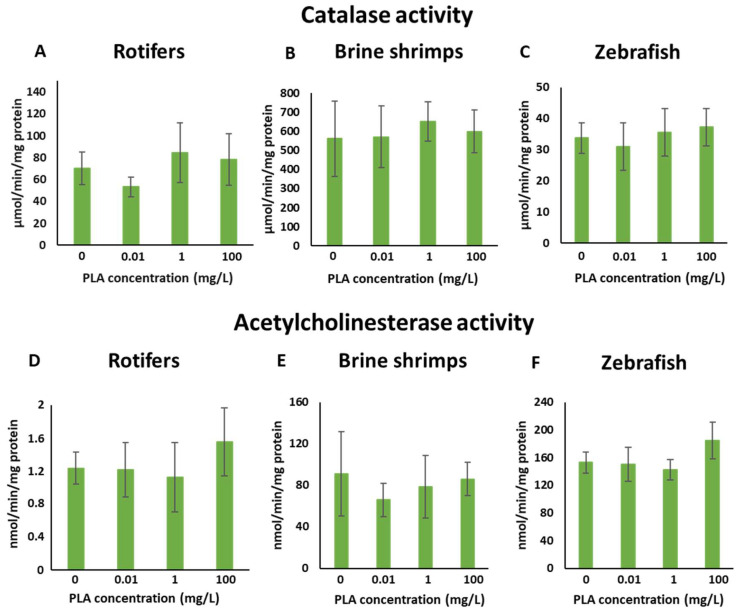
Biomarker responses in rotifers, brine shrimps and zebrafish embryos exposed to different concentrations of PLA NPs. (**A**–**C**) Catalase activity (µmol/min/mg prot). (**D**–**F**) Acetylcholinesterase activity (nmol/min/mg prot). Vertical segments show standard deviations.

Exposure to PLA NPs did not lead to significant changes in the activity levels of both AChE and catalase in either zooplankton species or zebrafish embryos (Figure 8) when compared to their respective controls. Additionally, no distinct trends were observed for both enzyme activities across the treated groups.

## 4. Discussion

Research on the toxicity of bio-based NPs to aquatic organisms remains limited, with only a few studies available compared to the more extensive body of knowledge on PLA MPs (Table 3). While PLA MPs have been tested across a wide range of invertebrates and fish species, investigations on PLA NPs are scarce and restricted to only a handful of taxa. Notably, none of the four PLA NP studies reported involved *Brachionus plicatilis* or *Artemia salina*, the model zooplankton organisms employed in the present study.

PLA NPs used in the present work showed a well-defined round shape and overall dispersion, although some aggregates could be observed at TEM. In contrast with the information available from the provider, the plain NPs were smaller than the fluorescent particles and showed a narrower size distribution. Both particle types displayed low zeta potential values when dispersed in salt water (0.88 ± 1.86 mV) and zebrafish embryo water (2.35 ± 0.95 mV) at the highest concentration of 100 mg/L used for the exposures. Nevertheless, hydrodynamic particle size did not vary significantly in salt water (266.94 ± 23.47 nm) and zebrafish embryo water (311.37 ± 27.58 nm) compared to the nominal size in pure deionised water (250 nm) according to the manufacturer’s information. The size and zeta potential of particles may significantly influence their adsorption, endocytic uptake, and subsequent particle toxicity [59]. Research on NPs of different compositions (such as silicon, silver, PS, or carbon) has shown that positively charged nanoparticles with a high zeta potential are more toxic than negatively charged ones [60,61,62]. This increased toxicity is generally attributed to positively charged nanoparticles’ higher ability to interact with cell membranes via attractive electrostatic interactions with negatively charged phospholipids or membrane proteins, resulting in increased cellular uptake, which can lead to oxidative stress, inflammation, genotoxicity, and cell death [63]. The low zeta potential values of PLA NPs used in this work may contribute to the observed lack of toxicity, as evidenced by the absence of significant mortality and biomarker alteration in our assays. This suggests limited interaction with biological membranes, which aligns with the low surface charge of the particles.

**Table 3:** Literature review on the studies addressing the toxicity of bio-based micro- and nanoplastics.

Material	Species	References
Hydroxymethylfurfural	*Daphnia magna*	[64]
Polyhydroxyalkanoate microbeads	*Nitokra lacustris pacifica*	[65]
Polyhydroxyalkanoates	*Streptomyces coelicolor*	[9]
Polyhydroxybutyrate	*Anabaena* sp. *Chlamydomonas reinhardtii* *Daphnia magna*	[66]
	*Hydra viridissima*	[67]
	*Lates calcarifer*	[68]
	*Gammarus fossarum*	[69]
Polyhydroxybutyrate (PHB) Polyhydroxyalkanoate copolymer (PHBVV)	*Artemia franciscana*	[70]
Poly(3-hydroxybutyrate-co-3-hydroxyhexanoate) (PHBH)	*Artemia salina*	[71]
PHBV and PLA leachates	*Aliivibrio fischeri* *Rhodomonas salina* *Paracentrotus lividus* *Mytilus galloprovincialis*	[72]
Polybutylene succinate-polybutyrate adipate terephthalate (PBS-PBAT) leachate	*Aliivibrio fischeri* *Oryzias latipes*	[73]
Poly(butylene adipate-co-terephthalate, PBAT)	*Danio rerio* (embryo and juvenile)	[74]
PLA MPs	*Daphnia magna*	[75,76,77,78]
	*Diaphanosoma celebensis*	[79]
	*Artemia franciscana*	[80,81]
	*Vibrio fischeri* *Phaedactylum tricornutum* *Brachionus plicatilis* *Tigriopus fulvus* *Corophium insidiorium* *Gammarus aequicauda* *Artemia franciscana*	[82]
	*Tigriopus japonicus*	[83]
	*Paracentrotus lividus*	[84,85,86]
	*Mytilus galloprovincialis*	[87]
	*Mytilus edulis*	[88,89]
	*Perna viridis*	[90]
	*Danio rerio* (larvae)	[91]
	*Danio rerio* (adults)	[92,93,94]
	*Lates calcarifer*	[95]
	*Clarias gariepinus*	[96]
	*Perca fluviatilis*	[97]
	*Oryzias melastigma*	[98]
	*Oreochromis mossambicus*	[99]
	*Carassius auratus*	[100]
PLA NPs	*Gammarus roeseli*	[101]
	*Hydra viridissima*	[102]
	*Danio rerio* (larvae)	[102,103]

The present study confirmed that fluorescent PLA NPs were uptaken by all tested species in an exposure concentration-dependent manner, consistent with previous research highlighting aquatic organisms’ ability to uptake NPs. Internal accumulation of NPs can interfere with physiological processes and behaviour, ultimately impairing key fitness traits such as survival, growth, and reproduction in contaminated ecosystems. However, exposure of rotifers to PLA NPs did not cause a significant increase in mortality, with the LC50 being higher than the concentrations tested, suggesting that the polymer is not toxic to these organisms, even though the small size of the particles. A similar result was observed in brine shrimp, with no significant effect on mortality. While there are contrasting results regarding the acute toxicity of both PLA NPs and PLA MPs to aquatic organisms, the findings of this study align with others that indicate that PLA MPs and NPs do not affect the mortality of some invertebrates. Di Giannantonio et al. [81] reported that PLA MPs (25–350 μm; 1–100 mg/L; 24 h) were found in the digestive system of brine shrimp, but mobility and survival were not affected. Götz et al. [101] observed that PLA NPs (200/500 nm; 4.19 and 419 ng/L; 14 d) did not affect the mortality of the amphipod *Gammarus roeseli*. Additionally, Tong et al. [83] reported that secondary PBAT/PLA NPs (10–40 μm; 20 and 500 mg/L; 48 h) did not affect the survival of the copepod *Tigriopus japonica*. Similar results were found for other polymer NPs, such as PS, which is one of the most studied petroleum-based polymers. Bergami et al. [104] observed that PS NPs (50 nm; 100 μg/mL; 48 h) caused no mortality in brine shrimp larvae. Likewise, the study of Martínez-Álvarez et al. [45] found that exposure to PS NPs (50 and 500 nm; 34 ng/L–6.68 mg/L; 48 h) did not cause a significant impact on brine shrimp survival.

In rotifers, our observations correspond with those reported by Snell and Hicks [105], who found that PS NPs (37–2980 nm; 28.7 or 2.87 μg/mL; 2–48 h) were readily ingested and predominantly localised within the intestinal tract. They noted that particles approximately 83 nm in size remained within the digestive system until elimination. Although Snell and Hicks [105] examined PS NPs rather than PLA NPs, the particle size ranges are comparable, and given the limited research on PLA NPs’ interactions with rotifers, their findings provide valuable context for comparison. Similarly, in brine shrimp, our results are in line with those of Di Giannantonio et al. [81], who documented the accumulation of PLA MPs (25–350 μm; 1–100 mg/L; 24 h) within the digestive system. Supporting this, Charoeythornkhajhornchai et al. [80] also reported observable accumulation of smaller PLA MPs (<60 μm; 6.5–100 μg/mL; 48 h) in brine shrimp after 24 h. For zebrafish larvae, a concentration-dependent uptake of fluorescent PLA NPs was observed, with nanoparticles predominantly localised in the gastrointestinal tract and eye regions. This trend is consistent with findings by Xu et al. [106], who showed that fluorescent PS MPs (500 nm; 0.1–10 mg/L; 120 h) accumulated around the embryonic chorion prior to hatching, and after hatching were internalised into the yolk sac, pericardium, and gastrointestinal tract. Similarly, Pitt et al. [15] demonstrated that PS NPs (100 nm; 0.1, 1 and 10 ppm; 48–120 h) accumulated in the yolk sac within 24 hpf and subsequently migrated to various internal organs, including the gastrointestinal tract, gallbladder, liver, pancreas, heart, and brain, during development.

One potential harmful consequence of plastic particle uptake through the digestive system is false satiation, where ingested particles reduce food intake, leading to decreased energy availability for vital biological functions. Changes in physiological functions and feeding behaviour are known indicators of sublethal effects of toxicants on test organisms. Although these effects may be mild, they can have a significant impact on the overall health and survival of the organisms. For the ingestion test in the zooplankton species, some studies assess the feeding or ingestion rate of organisms exposed to nanoparticles by quantifying the number of particles ingested [69,107] or food ingested [76,77,78,79,80,81,82,83,84,85,86,87,88,89,90,91,92,93,94,95,96,97,98,99,100,101,102,103,104,105,106,107,108] and not the capacity for ingestion, like in the case of this study. Reduced ingestion in the presence of stressors is an adaptive response to minimise exposure to poisonous substances [105]. The ingestion test in rotifers indicated no significant difference at the tested concentrations. The study by Snell and Hicks [105] showed a reduction in the feeding behaviour of *Brachionus manjavacas*, with reduced ingestion in response to exposure to PS NPs (50, 100, and 200 nm; 3 μg/mL; 45 min). This does not agree with our study; the difference in polymer type and size might explain the differences between the two studies, as PLA NPs do not appear to have a toxic effect, and the organism might not perceive it as poisonous, perhaps due to its bio-based origin.

The ingestion test developed for rotifers was also applied to brine shrimp, which displayed a significant dose-dependent reduction in feeding capacity, with the highest effect observed at the highest concentration. However, the EC50 value obtained was higher than the highest tested concentration. The presence of red microspheres in faecal matter indicates that feeding activity persisted during exposure, although the proportion of feeding individuals decreased by 26% at 100 mg/L compared to the control. This suggests that feeding was impaired but not entirely suppressed. This observation aligns with the findings of Savva et al. [76], where they reported significant inhibition in the post-exposure feeding rate of the crustacean *Daphnia magna* exposed to PLA MPs (<60 μm; 150 mg/L; 4–24 h). Similarly, Amelia et al. [65] documented the uptake and egestion of PHA (polyhydroxyalkanoate) microbeads (10.1–140 μm; ~700 microbeads/mL; 24 h) as faecal pellets by copepods *Nitokra lacustris*. Additionally, Bergami et al. [104] also noted the accumulation of PS NPs (50 nm; 100 μg/mL; 48 h) in the gut of brine shrimp and subsequent egestion as faecal pellets. A decrease in ingestion capacity is likely to impact an organism’s fitness, as reduced energy intake will negatively affect its ability to maintain optimal functioning and subsequently reproduction, resulting in an adverse effect on the population. In this study, the presence of red microspheres in faecal matter was only used as a tracer to confirm feeding activity. However, previous studies have suggested that if MPs and NPs are incorporated into faecal pellets, they may alter pellet density and sinking dynamics, with potential implications for vertical carbon flux and ocean carbon sequestration [109].

Early development is an important period in the growth of organisms, and disturbances by environmental stressors can have serious effects on development and adult life [110]. In this study, zebrafish embryos exposed to different concentrations of PLA NPs showed no significant effects on mortality, mean hatch time, and malformation rate. This aligns with a study by Tamayo-Belda et al. [102], where exposure to secondary PLA NPs (<1000 nm; 1 and 10 mg/L; 96 h) did not affect mortality, hatching time, or malformation rates, although a significant decrease in heartbeat rate was recorded. Similarly, Zhang et al. [111] found that exposure to virgin and degraded PLA MPs (5–50 μm; 0.1–25 mg/L; 96 h) did not significantly affect hatching, heart rate, deformity, or survival rate, demonstrating that PLA MPs exhibited no cardiotoxic or lethal effects on zebrafish larvae. However, Luan et al. [103] observed that PLA NPs and MPs (667.5–4213.5 nm; 100, 250, and 500 mg/L; 96 h) caused increased mortality, hatch time, malformation, and anxiety-like behaviour in zebrafish larvae and Zhang et al. [94] also reported that zebrafish embryos from females treated with both undegraded and degraded PLA MPs (100 μm; 5 mg/L; 5 weeks) exhibited high mortality rates and increased heart rates, although no differences were observed in hatch time and body length compared to the control.

Acridine orange staining revealed fewer apoptotic cells in the gastrointestinal tract of zebrafish larvae treated with 1 mg/L PLA NPs. The fluorescence intensity was not noticeably different from that of the control, suggesting that PLA NPs likely did not induce an increase in cell death. These results align with the findings of Zhang et al. [111], who reported that virgin PLA MPs (5–50 μm; 1–10 mg/L; 96 h) did not notably induce apoptosis in zebrafish larvae.

Biomarkers provide a valuable means for understanding the effects of exposure to environmental pollutants on biological systems [112]. They serve as early warning signs in detecting alterations in biological systems and are increasingly used in ecotoxicological studies. One of the well-known biomarkers to assess impaired neurotransmission capacity is the inhibition of AChE activity. It is widely used as a biomarker of neurotoxicity in humans and other animals, including invertebrates such as molluscs, nematodes, crustaceans, and echinoderms [113,114,115,116]. In both vertebrates and invertebrates, AChE breaks down the neurotransmitter acetylcholine into acetic acid and choline within the cholinergic synapses of the nervous system. Monitoring AChE activity is employed to evaluate the impacts of many environmental contaminants, such as organophosphate and carbamate insecticides, that cause the inhibition of this enzyme [117,118] and, as discussed below, it has already been used to assess the impact of MP and NP pollution. Environmental pollutants have been reported to trigger oxidative stress in organisms by activating mechanisms that generate free radicals, such as reactive oxygen species (ROS), which can oxidise several biomolecules in cells, causing DNA strand breakage, protein oxidation and lipid peroxidation, resulting in cell damage and death [119]. Together with the low molecular weight antioxidants, such as glutathione, antioxidant enzymes like catalase, superoxide dismutase, and glutathione peroxidase hunt free radicals and serve as the first line of defence against xenobiotic-induced oxidative stress [120]. As for other nanomaterials [121], oxidative stress has been described as the main mechanism of NP toxicity [122].

Exposure of rotifers, brine shrimp, and zebrafish larvae to PLA NPs did not significantly alter AChE or CAT activities, suggesting no evidence of neurotoxicity or oxidative stress under the tested conditions. These findings are consistent with Khalid et al. [89], who observed no effects of PLA MPs (0.8–10 μm; 10 and 100 μg/L; 8 d) on the same biomarkers in *Mytilus edulis*, indicating preserved antioxidant defence and neurotransmission. Nevertheless, responses to MPs and NPs are far from uniform. Some studies report stimulatory effects: for instance, Luangrath et al. [77] found elevated CAT activity in *D. magna* exposed to PLA MPs (47.38 ± 6.54 μm; 1 mg/L; 48 h), and Chagas et al. [92] documented increased AChE activity in adult zebrafish after PLA MP exposure (2.34 ± 0.07 μm; 2.5 and 5 mg/L; 30 d). Conversely, inhibitory effects have also been reported, such as reduced AChE activity in zebrafish larvae exposed to PLA MPs (2.34 μm; 3 and 9 mg/L; 5 days) [91] and suppressed CAT activity in zebrafish embryos and larvae exposed to petroleum-derived PS particles (500 nm; 0.1–10 mg/L; 6 d [59] and 10.11 ± 0.78 μm; 0.1–100 μg/L; 120 h [123]). These discrepancies highlight the context-dependency of responses, with variations likely driven by particle size, concentration, exposure duration, life stage, and polymer type. When considered alongside the literature, the present findings suggest that plain PLA NPs, at least within the tested size range and concentrations, do not induce measurable neurotoxic or oxidative stress effects in zooplankton or zebrafish larvae, providing further baseline evidence for the relatively low toxicity of plain PLA nanoparticles.

In conclusion, this study suggests that the commercial plain PLA NP formulation assayed in this work, which does not contain additives according to the information provided by the manufacturer, is non-toxic to zooplankton species (rotifers *B. plicatilis* and brine shrimp *A. salina*) and to the early developmental stage of zebrafish at the assessed concentrations and endpoints considered. Available studies demonstrate that the effects of bioplastic NPs can vary based on polymer type (virgin or degraded), size, surface charge, test species, developmental stage, exposure concentration, exposure duration, and endpoints assessed. Our results reinforce the increasingly recognised hypothesis that chemical additives added to plastics to improve their performance at final applications significantly contribute to their toxicity. Thereby suggesting a need to distinguish between the effects of polymer matrices and those of the associated additives in environmental risk assessments, which could inform the formulation of less toxic chemical additives in the future. PLA NPs used in the present study are the base polymer and, thus, their toxicity and environmental impact could be lower in comparison to the final plastic product in the market.

More research using model organisms from different trophic levels is recommended to better understand and systematically compare the impact of bioplastic polymers in aquatic ecosystems. In addition, studies incorporating behavioural parameters and physiological functions could help determine impacts at longer time scales. Future investigations should also build upon these findings by employing a broader suite of biomarkers and molecular approaches to elucidate the biological effects of bioplastics. The integration of omics-based techniques will provide additional means to uncover biochemical and metabolic pathways affected by exposure, offering deeper insights into the molecular mechanisms underpinning organismal responses to bio-based plastic contaminants. The hazards and risks posed by biobased MPs and NPs necessitate mandatory precautionary actions to restrict their spread and environmental contamination, preventing an escalation of the plastic menace caused by traditional plastics.

## Figures and Tables

**Table 2:** Prevalence of malformations in 120 hpf surviving zebrafish larvae exposed to PLA NPs. N/O: Malformations not observed.

PLA NP Concentration (mg/L)	Total Malformation Prevalence	Specific Malformations			
		Spinal Deformity	Yolk Sac Oedema	Pericardial Oedema	Curved Tail
0	6.67	N/O	3.34	3.34	3.33
0.01	3.33	N/O	N/O	N/O	3.33
0.1	10	3.33	6.67	6.67	N/O
1	6.67	3.33	3.34	3.34	N/O
10	6.67	3.33	N/O	N/O	3.34
100	16.67	3.34	N/O	N/O	13.33

## Data Availability

The original contributions presented in this study are included in the article/Supplementary Materials. Further inquiries can be directed to the corresponding author.

## Supplementary Materials

The following supporting information can be downloaded at: https://www.mdpi.com/article/10.3390/jox15060196/s1, Figure S1: Micrographs of rotifers and brine shrimps after the exposure to red microspheres for the ingestion test; Figure S2: Quantification of the fluorescence signal of the fluorescent PLA NPs accumulated in the three species; Table S1: Odds ratio (OR) values and their confidence intervals (CI) at 95% indicating the increased risk of mortality in both zooplankton species exposed to PLA NPs according to the binomial logistic regression; Table S2: Odds ratio (OR) values and their confidence intervals (CI) at 95% indicating the increased risk of ingestion ability impairment in both zooplankton species exposed to PLA NPs according to the binomial logistic regression; Table S3: Odds ratio (OR) values and their confidence intervals (CI) at 95% indicating the increased risk of mortality in zebrafish larvae exposed to PLA NPs according to the binomial logistic regression; Table S4: Odds ratio (OR) values and their confidence intervals (CI) at 95% indicating the increased risk of malformation in zebrafish larvae exposed to PLA NPs according to the binomial logistic regression. File S1: Original images of Figure 1A,B, Figure 2, Figure 3, Figure 6, Figure 7 and Figure S1.

## References

[1] Ainali N.M., Kalaronis D., Evgenidou E., Kyzas G.Z., Bobori D.C., Kaloyianni M., Yang X., Bikiaris D.N., Lambropoulou D.A. (2022). Do poly(lactic acid) microplastics instigate a threat? A perception for their dynamic towards environmental pollution and toxicity. Sci. Total Environ..

[2] Plastics Europe (2024). Plastics—The Fast Facts 2024. https://plasticseurope.org/knowledge-hub/plastics-the-fast-facts-2024/.

[3] Hartmann N.B., Hüffer T., Thompson R.C., Hassellöv M., Verschoor A., Daugaard A.E., Rist S., Karlsson T.M., Brennholt N., Cole M. (2019). Are we speaking the same language? Recommendations for a definition and categorization framework for plastic debris. Environ. Sci. Technol..

[4] Zettler E.R., Mincer T.J., Amaral-Zettler L.A. (2013). Life in the “Plastisphere”: Microbial communities on plastic marine debris. Environ. Sci. Technol..

[5] Shi X., Chen Z., Wei W., Chen J., Ni B.-J. (2023). Toxicity of micro/nanoplastics in the environment: Roles of plastisphere and eco-corona. Soil Environ. Health.

[6] García-Gómez J.C., Garrigós M., Garrigós J. (2021). Plastic as a Vector of Dispersion for Marine Species With Invasive Potential A Review. Front. Ecol. Evol..

[7] Rochman C.M., Tahir A., Williams S.L., Baxa D.V., Lam R., Miller J.T., Teh F.C., Werorilangi S., Teh S.J. (2015). Anthropogenic debris in seafood: Plastic debris and fibers from textiles in fish and bivalves sold for human consumption. Sci. Rep..

[8] Smith M., Love D.C., Rochman C.M., Neff R.A. (2018). Microplastics in seafood and the implications for human health. Curr. Environ. Health Rep..

[9] Liu X., Ahmad S., Ma J., Wang D., Tang J. (2023). Comparative study on the toxic effects of secondary nanoplastics from biodegradable and conventional plastics on Streptomyces coelicolor M145. J. Hazard. Mater..

[10] Nolte T.M., Hartmann N.B., Kleijn J.M., Garnæs J., van de Meent D., Jan Hendriks A., Baun A. (2017). The toxicity of plastic nanoparticles to green algae as influenced by surface modification, medium hardness and cellular adsorption. Aquat. Toxicol..

[11] Li Z., Feng C., Wu Y., Guo X. (2020). Impacts of nanoplastics on bivalve: Fluorescence tracing of organ accumulation, oxidative stress and damage. J. Hazard. Mater..

[12] Yeo I.-C., Shim K.-Y., Kim K., Jeong C.-B. (2023). Maternal exposure to nanoplastic induces transgenerational toxicity in the offspring of rotifer *Brachionus koreanus*. Comp. Biochem. Physiol. Part C Toxicol. Pharmacol..

[13] Dong X., Liu X., Hou Q., Wang Z. (2023). From natural environment to animal tissues: A review of microplastics(nanoplastics) translocation and hazards studies. Sci. Total Environ..

[14] Li Y., Du X., Liu Z., Zhang M., Huang Y., Tian J., Jiang Q., Zhao Y. (2021). Two genes related to reproductive development in the juvenile prawn, *Macrobrachium nipponense*: Molecular characterization and transcriptional response to nanoplastic exposure. Chemosphere.

[15] Pitt J.A., Kozal J.S., Jayasundara N., Massarsky A., Trevisan R., Geitner N., Wiesner M., Levin E.D., Di Giulio R.T. (2018). Uptake, tissue distribution, and toxicity of polystyrene nanoparticles in developing zebrafish (*Danio rerio*). Aquat. Toxicol..

[16] Duan J., Li Y., Gao J., Cao R., Shang E., Zhang W. (2022). ROS-mediated photoaging pathways of nano- and micro-plastic particles under UV irradiation. Water Res..

[17] Fan X., Wei X., Hu H., Zhang B., Yang D., Du H., Zhu R., Sun X., Oh Y., Gu N. (2022). Effects of oral administration of polystyrene nanoplastics on plasma glucose metabolism in mice. Chemosphere.

[18] Lin W., Jiang R., Hu S., Xiao X., Wu J., Wei S., Xiong Y., Ouyang G. (2019). Investigating the toxicities of different functionalized polystyrene nanoplastics on *Daphnia magna*. Ecotoxicol. Environ. Saf..

[19] Woo J.-H., Seo H.J., Lee J.-Y., Lee I., Jeon K., Kim B., Lee K. (2023). Polypropylene nanoplastic exposure leads to lung inflammation through p38-mediated NF-κB pathway due to mitochondrial damage. Part. Fibre Toxicol..

[20] Vagner M., Boudry G., Courcot L., Vincent D., Dehaut A., Duflos G., Huvet A., Tallec K., Zambonino-Infante J.L. (2022). Experimental evidence that polystyrene nanoplastics cross the intestinal barrier of European seabass. Environ. Int..

[21] Zhou W., Tong D., Tian D., Yu Y., Huang L., Zhang W., Yu Y., Lu L., Zhang X., Pan W. (2023). Exposure to Polystyrene Nanoplastics Led to Learning and Memory Deficits in Zebrafish by Inducing Oxidative Damage and Aggravating Brain Aging. Adv. Healthc. Mater..

[22] European Bioplastics (2024). What Are Bioplastics?. https://www.european-bioplastics.org/bioplastics/.

[23] Di Bartolo A., Infurna G., Dintcheva N.T. (2021). A review of bioplastics and their adoption in the circular economy. Polymers.

[24] Naser A.Z., Deiab I., Darras B.M. (2021). Poly (lactic acid)(PLA) and polyhydroxyalkanoates (PHAs), green alternatives to petroleum-based plastics: A review. RSC Adv..

[25] European Bioplastic (2024). Bioplastics Market Development Update 2024. https://www.european-bioplastics.org/bioplastics-market-development-update-2024/.

[26] Ali S.S., Abdelkarim E.A., Elsamahy T., Al-Tohamy R., Li F., Kornaros M., Zuorro A., Zhu D., Sun J. (2023). Bioplastic production in terms of life cycle assessment: A state-of-the-art review. Environ. Sci. Ecotechnol..

[27] Tuominen J., Kylmä J., Kapanen A., Venelampi O., Itävaara M., Seppälä J. (2002). Biodegradation of lactic acid based polymers under controlled composting conditions and evaluation of the ecotoxicological impact. Biomacromolecules.

[28] Karava V., Siamidi A., Vlachou M., Christodoulou E., Zamboulis A., Bikiaris D.N., Kyritsis A., Klonos P.A. (2021). Block copolymers based on poly(butylene adipate) and poly(l-lactic acid) for biomedical applications: Synthesis, structure and thermodynamical studies. Soft Matter.

[29] Perin D., Rigotti D., Fredi G., Papageorgiou G.Z., Bikiaris D.N., Dorigato A. (2021). Innovative bio-based poly(lactic acid)/poly(alkylene furanoate)s fiber blends for sustainable textile applications. J. Polym. Environ..

[30] Fredi G., Rigotti D., Bikiaris D.N., Dorigato A. (2021). Tuning thermo-mechanical properties of poly(lactic acid) films through blending with bioderived poly(alkylene furanoate)s with different alkyl chain length for sustainable packaging. Polymer.

[31] Chrysafi I., Ainali N.M., Bikiaris D.N. (2021). Thermal degradation mechanism and decomposition kinetic studies of poly(lactic acid) and its copolymers with poly(hexylene succinate). Polymers.

[32] Terzopoulou Z., Zamboulis A., Bikiaris D.N., Valera M.A., Mangas A. (2021). Synthesis, properties, and enzymatic hydrolysis of poly (lactic acid)-co-poly (propylene adipate) block copolymers prepared by reactive extrusion. Polymers.

[33] Sanusi O.M., Benelfellah A., Papadopoulos L., Terzopoulou Z., Bikiaris D.N., Hocine N.A. (2021). Properties of poly(lactic acid)/montmorillonite/carbon nanotubes nanocomposites: Determination of percolation threshold. J. Mater. Sci..

[34] Tarani E., Črešnar K.P., Zemljič L.F., Chrissafis K., Papageorgiou G.Z., Lambropoulou D., Zamboulis A., Bikiaris D.N., Terzopoulou Z. (2021). Cold crystallization kinetics and thermal degradation of PLA composites with metal oxide nanofillers. Appl. Sci..

[35] Chen M., Chen F., Li Z., Haider M.R., Wei J., Chen G., Wang W., Wang J. (2023). Environmental risk assessment of microplastics and nanoplastics generated from biodegradable plastics in marine ecosystem. TrAC Trends Anal. Chem..

[36] Ubeda S., Aznar M., Alfaro P., Nerín C. (2019). Migration of oligomers from a food contact biopolymer based on polylactic acid (PLA) and polyester. Anal. Bioanal. Chem..

[37] Dahms H.-U., Hagiwara A., Lee J.-S. (2011). Ecotoxicology, ecophysiology, and mechanistic studies with rotifers. Aquat. Toxicol..

[38] Hagiwara A., Suga K., Akazawa A., Kotani T., Sakakura Y. (2007). Development of rotifer strains with useful traits for rearing fish larvae. Aquaculture.

[39] Kaneko G., Yoshinaga T., Yanagawa Y., Kinoshita S., Tsukamoto K., Watabe S. (2005). Molecular characterization of Mn-superoxide dismutase and gene expression studies in dietary restricted *Brachionus plicatilis* rotifers. Hydrobiologia.

[40] Zhu B., Zhu S., Li J., Hui X., Wang G.-X. (2018). The developmental toxicity, bioaccumulation and distribution of oxidized single walled carbon nanotubes in *Artemia salina*. Toxicol. Res..

[41] Arulvasu C., Jennifer S.M., Prabhu D., Chandhirasekar D. (2014). Toxicity effect of silver nanoparticles in brine shrimp *Artemia*. Sci. World J..

[42] Ates M., Daniels J., Arslan Z., Farah I.O., Rivera H.F. (2013). Comparative evaluation of impact of Zn and ZnO nanoparticles on brine shrimp (*Artemia salina*) larvae: Effects of particle size and solubility on toxicity. Environ. Sci. Process Impacts.

[43] Wang W., Gao H., Jin S., Li R., Na G. (2019). The ecotoxicological effects of microplastics on aquatic food web, from primary producer to human: A review. Ecotoxicol. Environ. Saf..

[44] Batel A., Borchert F., Reinwald H., Erdinger L., Braunbeck T. (2018). Microplastic accumulation patterns and transfer of benzo[a]pyrene to adult zebrafish (*Danio rerio*) gills and zebrafish embryos. Environ. Pollut..

[45] Martínez-Álvarez I., Le Menach K., Devier M.H., Cajaraville M.P., Budzinski H., Orbea A. (2022). Screening of the toxicity of polystyrene nano- and microplastics alone and in combination with benzo(a)pyrene in brine shrimp larvae and zebrafish embryos. Nanomaterials.

[46] Trevisan R., Voy C., Chen S., Di Giulio R.T. (2019). Nanoplastics decrease the toxicity of a complex PAH mixture but impair mitochondrial energy production in developing zebrafish. Environ. Sci. Technol..

[47] Bhagat J., Zang L., Nishimura N., Shimada Y. (2020). Zebrafish: An emerging model to study microplastic and nanoplastic toxicity. Sci. Total Environ..

[48] (2016). Water Quality—Determination of the Acute Toxicity to the Marine Rotifer Brachionus Plicatilis.

[49] Snell T.W. (2005). Rotifer Ingestion Test for Rapid Assessment of Toxicity. Small-Scale Freshwater Toxicity Investigations: Toxicity Test Methods.

[50] Leusch F.D.L., Ziajahromi S. (2021). Converting mg/L to Particles/L: Reconciling the Occurrence and Toxicity Literature on Microplastics. Environ. Sci. Technol..

[51] OECD (2013). 236: Fish Embryo Acute Toxicity (FET) Test. OECD Guidelines for the Testing of Chemicals.

[52] Beckman S. (2017). Using Acridine Orange to Measure Cell Death in Ethanol Treated Zebrafish Embryos. Biotek Application Note.

[53] Johann S., Nüßer L., Goßen M., Hollert H., Seiler T.B. (2020). Differences in biomarker and behavioral responses to native and chemically dispersed crude and refined fossil oils in zebrafish early life stages. Sci. Total Environ..

[54] Aebi H. (1984). Catalase In Vitro. Methods in Enzymology.

[55] Ellman G.L., Courtney K.D., Andres V., Featherstone R.M. (1961). A new and rapid colorimetric determination of acetylcholinesterase activity. Biochem. Pharmacol..

[56] Orbea A., González-Soto N., Lacave J.M., Barrio I., Cajaraville M.P. (2017). Developmental and reproductive toxicity of PVP/PEI-coated silver nanoparticles to zebrafish. Comp. Biochem. Physiol. Part C Toxicol. Pharmacol..

[57] Firth D. (1993). Bias Reduction of Maximum Likelihood Estimates. Biometrika.

[58] Kosmidis I. BRGLM: Bias Reduction in Binomial-Response Generalized Linear Models. https://cran.r-project.org/web/packages/brglm/brglm.pdf.

[59] Suman A., Mahapatra A., Gupta P., Ray S.S., Singh R.K. (2023). Polystyrene microplastics modulated bdnf expression triggering neurotoxicity via apoptotic pathway in zebrafish embryos. Comp. Biochem. Physiol. Part C Toxicol. Pharmacol..

[60] Kim J., Chankeshwara S.V., Thielbeer F., Jeong J., Donaldson K., Bradley M., Cho W.-S. (2016). Surface charge determines the lung inflammogenicity: A study with polystyrene nanoparticles. Nanotoxicology.

[61] Li R., Wang X., Ji Z., Sun B., Zhang H., Chang C.H., Lin S., Meng H., Liao Y.-P., Wang M. (2013). Surface charge and cellular processing of covalently functionalized multiwall carbon nanotubes determine pulmonary toxicity. ACS Nano.

[62] Shahbazi M.-A., Hamidi M., Mäkilä E.M., Zhang H., Almeida P.V., Kaasalainen M., Salonen J.J., Hirvonen J.T., Santos H.A. (2013). The mechanisms of surface chemistry effects of mesoporous silicon nanoparticles on immunotoxicity and biocompatibility. Biomaterials.

[63] Mahmoudi M., Lynch I., Ejtehadi M.R., Monopoli M.P., Bombelli F.B., Laurent S. (2011). Protein− nanoparticle interactions: Opportunities and challenges. Chem. Rev..

[64] Swart E., de Boer T.E., Chen G., Vooijs R., van Gestel C.A., van Straalen N.M., Roelofs D. (2019). Species-specific transcriptomic responses in *Daphnia magna* exposed to a bio-plastic production intermediate. Environ. Pollut..

[65] Amelia T.S.M., Sukri S.N.F., Nursabrina A., Jaapar R., Amin M., Bhubalan K. (2020). Uptake and egestion of polyhydroxyalkanoate microbeads. J. Sustain. Sci. Manag..

[66] González-Pleiter M., Tamayo-Belda M., Pulido-Reyes G., Amariei G., Leganés F., Rosal R., Fernández-Piñas F. (2019). Secondary nanoplastics released from a biodegradable microplastic severely impact freshwater environments. Environ. Sci. Nano.

[67] Santos A., Oliveira M., Almeida M., Lopes I., Venâncio C. (2024). Short- and long-term toxicity of nano-sized polyhydroxybutyrate to the freshwater cnidarian *Hydra viridissima*. Sci. Total Environ..

[68] Sai S., Mani R., Vijayakumar P., Ganesan M., Velu K., Ayyamperumal R., Rajagopal R., Chang S.W., Alfarhan A., Ravindran B. (2022). Risk assessment of potential toxicity induced by bio and synthetic plastic microspheres in *Lates calcarifer*. Chemosphere.

[69] Straub S., Hirsch P.E., Burkhardt-Holm P. (2017). Biodegradable and petroleum-based microplastics do not differ in their ingestion and excretion but in their biological effects in a freshwater invertebrate *Gammarus fossarum*. Int. J. Environ. Res. Public Health.

[70] Tanadchangsaeng N., Pattanasupong A. (2022). Evaluation of Biodegradabilities of Biosynthetic Polyhydroxyalkanoates in Thailand Seawater and Toxicity Assessment of Environmental Safety Levels. Polymers.

[71] Ibarretxe J., Alonso L., Aranburu N., Guerrica-Echevarría G., Orbea A., Iturrondobeitia M. (2022). Sustainable PHBH–Alumina Nanowire Nanocomposites: Properties and Life Cycle Assessment. Polymers.

[72] Laranjeiro F., Rotander A., López-Ibáñez S., Vilas A., Södergren Seilitz F., Clérandeau C., Sampalo M., Rial D., Bellas J., Cachot J. (2024). Comparative assessment of the acute toxicity of commercial bio-based polymer leachates on marine plankton. Sci. Total Environ..

[73] Dusacre E., Le Picard C., Hausard V., Rigolet C., Ekoja F., Jean M., Clérandeau C., Villette S., Lagarde F., Lecomte S. (2025). Distinct toxicity profiles of conventional and biodegradable fishing nets’ leachates after artificial aging. J. Hazard. Mater..

[74] Xie M., Cai K., Zhang J., Tu S., Feng J. (2024). Preparation of PBAT microplastics and their potential toxicity to zebrafish embryos and juveniles. Aquat. Toxicol..

[75] An G., Na J., Song J., Jung J. (2024). Chronic toxicity of biodegradable microplastic (Polylactic acid) to *Daphnia magna*: A comparison with polyethylene terephthalate. Aquat. Toxicol..

[76] Savva K., Farré M., Barata C. (2023). Sublethal effects of bio-plastic microparticles and their components on the behaviour of *Daphnia magna*. Environ. Res..

[77] Luangrath A., Na J., Kalimuthu P., Song J., Kim C., Jung J. (2024). Ecotoxicity of polylactic acid microplastic fragments to *Daphnia magna* and the effect of ultraviolet weathering. Ecotoxicol. Environ. Saf..

[78] Zimmermann L., Göttlich S., Oehlmann J., Wagner M., Völker C. (2020). What are the drivers of microplastic toxicity? Comparing the toxicity of plastic chemicals and particles to *Daphnia magna*. Environ. Pollut..

[79] Ali W., Jeong H., Tisn M.L., Favrelle-Huret A., Thielemans W., Zinck P., Souissi S., Lee J.-S. (2024). The comparative toxicity of biobased, modified biobased, biodegradable, and petrochemical-based microplastics on the brackish water flea *Diaphanosoma celebensis*. Sci. Total Environ..

[80] Charoeythornkhajhornchai P., Kunjiek T., Chaipayang S., Phosri S. (2023). Toxicity assessment of bioplastics on brine shrimp (*Artemia franciscana*) and cell lines. Emerg. Contam..

[81] Di Giannantonio M., Gambardella C., Miroglio R., Costa E., Sbrana F., Smerieri M., Carraro G., Utzeri R., Faimali M., Garaventa F. (2022). Ecotoxicity of Polyvinylidene Difluoride (PVDF) and Polylactic Acid (PLA) microplastics in marine zooplankton. Toxics.

[82] Manfra L., Albarano L., Rotini A., Biandolino F., Prato E., Carraturo F., Chiaretti G., Faraponova O., Salamone M., Sebbio C. (2025). Can biodegradable plastics mitigate plastamination? Feedbacks from marine organisms. J. Hazard. Mater..

[83] Tong H., Zhong X., Duan Z., Yi X., Cheng F., Xu W., Yang X. (2022). Micro- and nanoplastics released from biodegradable and conventional plastics during degradation: Formation, aging factors, and toxicity. Sci. Total Environ..

[84] Quade J., López-Ibáñez S., Beiras R. (2022). Mesocosm trials reveal the potential toxic risk of degrading bioplastics to marine life. Mar. Pollut. Bull..

[85] Uribe-Echeverría T., Beiras R. (2022). Acute toxicity of bioplastic leachates to *Paracentrotus lividus* sea urchin larvae. Mar. Environ. Res..

[86] Viel T., Cocca M., Manfra L., Caramiello D., Libralato G., Zupo V., Costantini M. (2023). Effects of biodegradable-based microplastics in *Paracentrotus lividus* Lmk embryos: Morphological and gene expression analysis. Environ. Pollut..

[87] Capolupo M., Rafiq A., Coralli I., Alessandro T., Valbonesi P., Fabbri D., Fabbri E. (2023). Bioplastic leachates characterization and impacts on early larval stages and adult mussel cellular, biochemical and physiological responses. Environ. Pollut..

[88] Green D.S., Colgan T.J., Thompson R.C., Carolan J.C. (2019). Exposure to microplastics reduces attachment strength and alters the haemolymph proteome of blue mussels (*Mytilus edulis*). Environ. Pollut..

[89] Khalid A., Zalouk-Vergnoux A., Benali S., Mincheva R., Raquez J.-M., Bertrand S., Poirier L. (2021). Are bio-based and biodegradable microplastics impacting for blue mussel (*Mytilus edulis*)?. Mar. Pollut. Bull..

[90] Joyce P.W.S., Falkenberg L.J. (2022). Microplastics, both non-biodegradable and biodegradable, do not affect the whole organism functioning of a marine mussel. Sci. Total Environ..

[91] de Oliveira J.P.J., Estrela F.N., Rodrigues A.S.d.L., Guimarães A.T.B., Rocha T.L., Malafaia G. (2021). Behavioral and biochemical consequences of *Danio rerio* larvae exposure to polylactic acid bioplastic. J. Hazard. Mater..

[92] Chagas T.Q., Freitas Í.N., Montalvão M.F., Nobrega R.H., Machado M.R.F., Charlie-Silva I., Araújo APd C., Guimarães A.T.B., Alvarez TGd S., Malafaia G. (2021). Multiple endpoints of polylactic acid biomicroplastic toxicity in adult zebrafish (*Danio rerio*). Chemosphere.

[93] Duan Z., Cheng H., Duan X., Zhang H., Wang Y., Gong Z., Zhang H., Sun H., Wang L. (2022). Diet preference of zebrafish (*Danio rerio*) for bio-based polylactic acid microplastics and induced intestinal damage and microbiota dysbiosis. J. Hazard. Mater..

[94] Zhang L., Luo Y., Zhang Z., Pan Y., Li X., Zhuang Z., Li J., Luo Q., Chen X. (2024). Enhanced reproductive toxicity of photodegraded polylactic acid microplastics in zebrafish. Sci. Total Environ..

[95] Xie M., Xu P., Zhou W., Xu X., Li H., He W., Yue W., Zhang L., Ding D., Suo A. (2022). Impacts of conventional and biodegradable microplastics on juvenile *Lates calcarifer*: Bioaccumulation, antioxidant response, microbiome, and proteome alteration. Mar. Pollut. Bull..

[96] Jang F.H., Wong C., Choo J., Aun Sia E.S., Mujahid A., Müller M. (2022). Increased transfer of trace metals and *Vibrio sp*. from biodegradable microplastics to catfish *Clarias gariepinus*. Environ. Pollut..

[97] König Kardgar A., Ghosh D., Sturve J., Agarwal S., Carney Almroth B. (2023). Chronic poly(l-lactide) (PLA)- microplastic ingestion affects social behavior of juvenile European perch (*Perca fluviatilis*). Sci. Total Environ..

[98] Wen S., Yin X., Zhang Y., Diao X. (2024). Chronic exposure to low concentrations of microplastics causing gut tissue damage but non-significant changes in the microbiota of marine medaka larvae (*Oryzias melastigma*). Mar. Environ. Res..

[99] Bao R., Cheng Z., Peng L., Mehmood T., Gao L., Zhuo S., Wang L., Su Y. (2023). Effects of biodegradable and conventional microplastics on the intestine, intestinal community composition, and metabolic levels in tilapia (*Oreochromis mossambicus*). Aquat. Toxicol..

[100] Khosrovyan A., Melkonyan H., Rshtuni L., Gabrielyan B., Kahru A. (2023). Polylactic Acid-Based Microplastic Particles Induced Oxidative Damage in Brain and Gills of Goldfish *Carassius auratus*. Water.

[101] Götz A., Beggel S., Geist J. (2022). Dietary exposure to four sizes of spherical polystyrene, polylactide and silica nanoparticles does not affect mortality, behaviour, feeding and energy assimilation of *Gammarus roeseli*. Ecotoxicol. Environ. Saf..

[102] Tamayo-Belda M., Venâncio C., Fernandez-Piñas F., Rosal R., Lopes I., Oliveira M. (2023). Effects of petroleum-based and biopolymer-based nanoplastics on aquatic organisms: A case study with mechanically degraded pristine polymers. Sci. Total Environ..

[103] Luan J., Zhang S., Xu Y., Wen L., Feng X. (2023). Effects of microplastic exposure on the early developmental period and circadian rhythm of zebrafish (*Danio rerio*): A comparative study of polylactic acid and polyglycolic acid. Ecotoxicol. Environ. Saf..

[104] Bergami E., Bocci E., Vannuccini M.L., Monopoli M., Salvati A., Dawson K.A., Corsi I. (2016). Nano-sized polystyrene affects feeding, behavior and physiology of brine shrimp *Artemia franciscana* larvae. Ecotoxicol. Environ. Saf..

[105] Snell T.W., Hicks D.G. (2011). Assessing toxicity of nanoparticles using Brachionus manjavacas (Rotifera). Environ. Toxicol..

[106] Xu C., Guo H., Wang R., Li T., Gu L., Sun L. (2021). Accumulation and Distribution of Fluorescent Microplastics in the Early Life Stages of Zebrafish. Journal of visualized experiments. JoVE.

[107] Au S.Y., Bruce T.F., Bridges W.C., Klaine S.J. (2015). Responses of *Hyalella azteca* to acute and chronic microplastic exposures. Environ. Toxicol. Chem..

[108] Xuan L., Lin L., Shaoguo R., Junho E., Dong W., Samreen, Jun W. (2023). Nanoplastics induce more severe multigenerational life-history trait changes and metabolic responses in marine rotifer *Brachionus plicatilis*: Comparison with microplastics. J. Hazard. Mater..

[109] Cole M., Lindeque P., Fileman E., Halsband C., Goodhead R., Moger J., Galloway T.S. (2013). Microplastic ingestion by zooplankton. Environ. Sci. Technol..

[110] Schweizer M., Dieterich A., Corral Morillas N., Dewald C., Miksch L., Nelson S., Wick A., Triebskorn R., Köhler H.-R. (2018). The importance of sediments in ecological quality assessment of stream headwaters: Embryotoxicity along the Nidda River and its tributaries in Central Hesse, Germany. Environ. Sci. Eur..

[111] Zhang X., Xia M., Su X., Yuan P., Li X., Zhou C., Wan Z., Zou W. (2021). Photolytic degradation elevated the toxicity of polylactic acid microplastics to developing zebrafish by triggering mitochondrial dysfunction and apoptosis. J. Hazard. Mater..

[112] Travis C.C. (2013). Use of Biomarkers in Assessing Health and Environmental Impacts of Chemical Pollutants.

[113] Forget J., Beliaeff B., Bocquene G. (2003). Acetylcholinesterase activity in copepods (*Tigriopus brevicornis*) from the Vilaine River estuary, France, as a biomarker of neurotoxic contaminants. Aquat. Toxicol..

[114] Gaitonde D., Sarkar A., Kaisary S., Silva C.D., Dias C., Rao D.P., Ray D., Nagarajan R., De Sousa S.N., Sarker S. (2006). Acetylcholinesterase activities in marine snail (*Cronia contracta*) as a biomarker of neurotoxic contaminants along the Goa coast, West coast of India. Ecotoxicology.

[115] Matozzo V., Tomei A., Marin M.G. (2005). Acetylcholinesterase as a biomarker of exposure to neurotoxic compounds in the clam *Tapes philippinarum* from the Lagoon of Venice. Mar. Pollut. Bull..

[116] Prüst M., Meijer J., Westerink R.H. (2020). The plastic brain: Neurotoxicity of micro-and nanoplastics. Part. Fibre Toxicol..

[117] Fu H., Xia Y., Chen Y., Xu T., Xu L., Guo Z., Xu H., Xie H.Q., Zhao B. (2018). Acetylcholinesterase is a potential biomarker for a broad spectrum of organic environmental pollutants. Environ. Sci. Technol..

[118] Tufi S., Leonards P., Lamoree M., de Boer J., Legler J., Legradi J. (2016). Changes in neurotransmitter profiles during early zebrafish (*Danio rerio*) development and after pesticide exposure. Environ. Sci. Technol..

[119] Li S., Tan H.Y., Wang N., Zhang Z.J., Lao L., Wong C.W., Feng Y. (2015). The Role of oxidative stress and antioxidants in liver diseases. Int. J. Mol. Sci..

[120] Cajaraville M.P., Hauser L., Carvalho G., Hylland K., Olabarrieta I., Lawrence A.J., Lowe D., Goksøyr A., Lawrence A.J., Hemingway K.L. (2003). Genetic Damage and the Molecular/Cellular Response to Pollution. Effects of Pollution in Fish.

[121] Massarsky A., Trudeau V.L., Moon T.W. (2014). Predicting the environmental impact of nanosilver. Environ. Toxicol. Pharmacol..

[122] Jeong J., Im J., Choi J. (2024). Integrating aggregate exposure pathway and adverse outcome pathway for micro/nanoplastics: A review on exposure, toxicokinetics, and toxicity studies. Ecotoxicol. Environ. Saf..

[123] Ding P., Xiang C., Li X., Chen H., Shi X., Li X., Huang C., Yu Y., Qi J., Li A.J. (2023). Photoaged microplastics induce neurotoxicity via oxidative stress and abnormal neurotransmission in zebrafish larvae (*Danio rerio*). Sci. Total Environ..

