# Supplementary material for: PLA Nanoplastics Accumulate but Do Not Cause Acute Toxicity to Marine Rotifers, Brine Shrimps, and Zebrafish Embryos
Source: J Xenobiot. 2025 Nov 12;15(6):196. doi: 10.3390/jox15060196 (PMC12641657; doi:10.3390/jox15060196)
Supplement: Supplementary file 1 [file jox-15-00196-s001.zip › jox-3945975-supplementary-done-proof-done-send XML.pdf]

Journal of Xenobiotics

Supplementary Materials: PLA Nanoplastics Accumulate but Do Not Cause Acute Toxicity to Marine Rotifers, Brine Shrimps, and Zebrafish Embryos

Doyinsola Suliat Mustapha, Olga Rodríguez-Díaz, Miren P. Cajaraville and Amaia Orbea

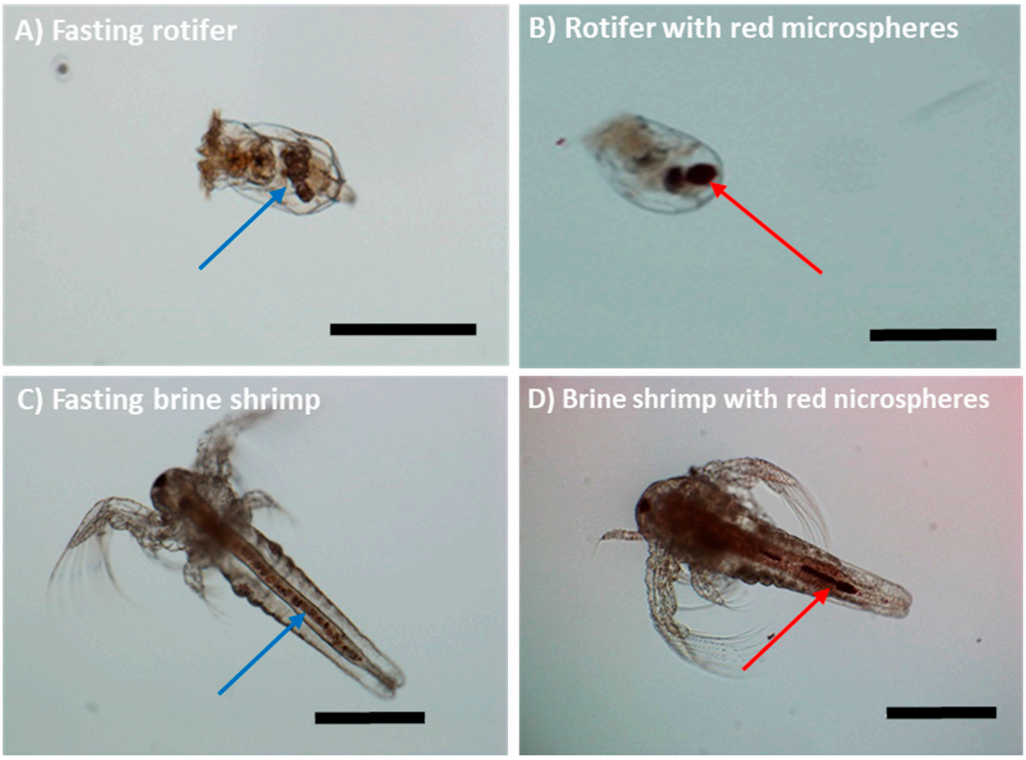

Figure S1: Micrographs of rotifers and brine shrimps after the exposure to red microspheres for the ingestion test. (a) Rotifer showing no ingestion of red microspheres; (b) Rotifer that ingested red microspheres; (c) Brine shrimp showing no ingestion of red microspheres; (d) Brine shrimp that ingested red microspheres. Empty guts are indicated by blue arrows and guts containing red microspheres are indicated by red arrows. Scale bars A, C, D: 100 µm. Scale bar B: 50 µm.

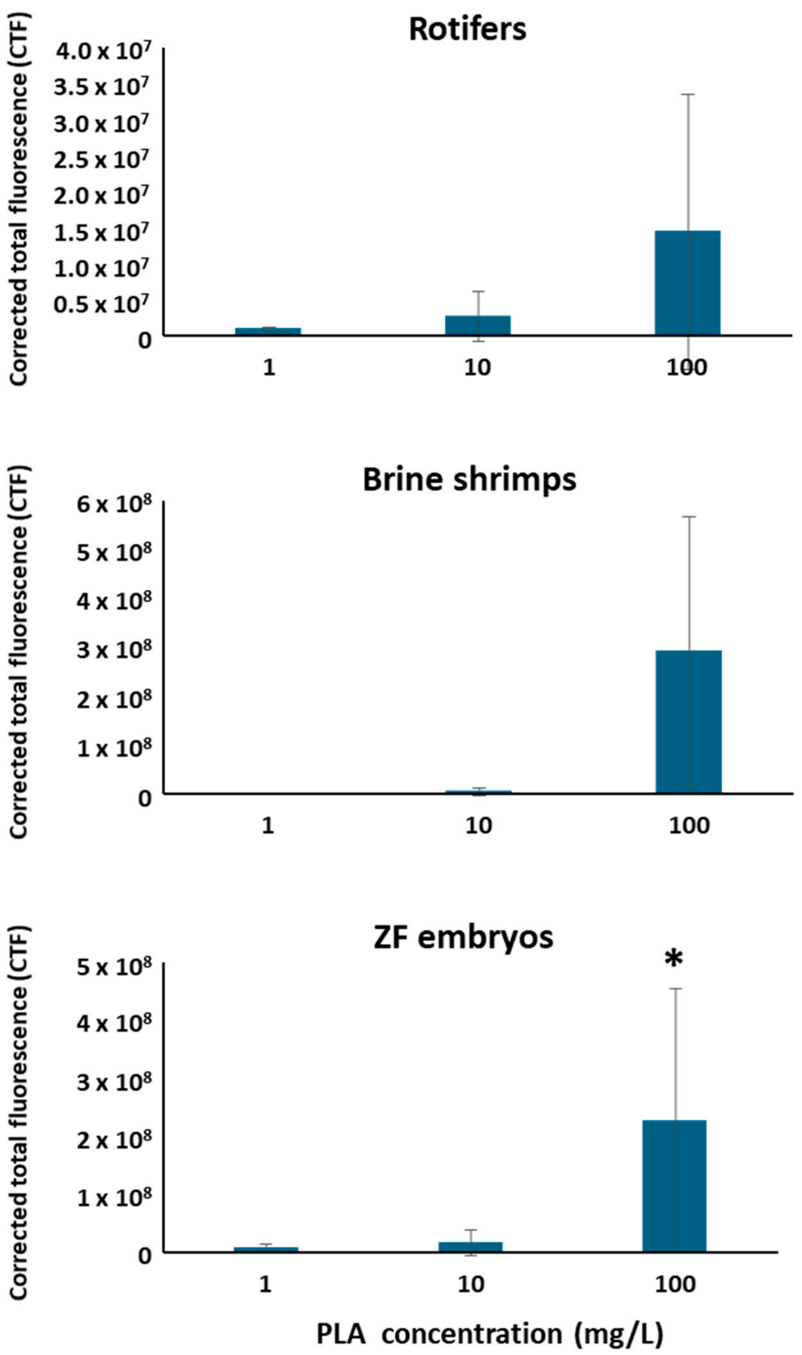

Figure S2: Quantification of the fluorescence signal of the fluorescent PLA NPs accumulated in the three species. Measurements were done in 3-4 individuals per exposure group. As it can be observed in the three cases, there is an exposure concentration-dependent increase of the fluorescence signal, although variability among individuals was high. Statistical significant results (asterisk) were only detected for zebrafish embryos exposed to 100 mg/L compared to the other exposure concentrations.

The following tables show the results of the statistical analyses carried out with the bioassay data. The tables contain the values of the odds ratios (and their confidence intervals at 95%) derived from the binomial logistic regression and show the risk under exposure to a given concentration of the PLA NPs in comparison to its respective control group.

Table S1: Odds ratio (OR) values and their confidence intervals (CI) at 95% indicating the increased risk of mortality in both zooplankton species exposed to PLA NPs according to the binomial logistic regression.

	**Rotifers**				**Brine shrimps**			
	**24 hours**		**48 hours**		**24 hours**		**48 hours**	
**mg/L**	**OR**	**CI 95%**	**OR**	**CI 95%**	**OR**	**CI 95%**	**OR**	**CI 95%**
**0.01**	3.81	0.29 – Inf	1.67	-0.73 – 1.87	1.00	-Inf – Inf	1.55	-0.54 – Inf
**0.1**	2.62	-0.11 – Inf	2.33	-0.34 – 2.16	2.18	-1.28 – Inf	8.00	-1.28 – Inf
**1**	3.22	0.13 – Inf	1.73	-0.65 – 1.88	1.00	-Inf – Inf	1.00	-Inf – Inf
**10**	1.01	-1.19 – Inf	1.98	-0.57 – 2.04	1.00	-Inf – Inf	1.00	-Inf – Inf
**100**	9.25	-1.28 – Inf	2.21	-0.42 – 2.13	1.00	-Inf – Inf	1.00	-Inf – Inf

Table S2: Odds ratio (OR) values and their confidence intervals (CI) at 95% indicating the increased risk of ingestion ability impairment in both zooplankton species exposed to PLA NPs according to the binomial logistic regression. Asterisks indicate significant differences compared to the corresponding control ($p<0.05$).

	**Rotifers**		**Brine shrimps**	
**mg/L**	**OR**	**CI 95%**	**OR**	**CI 95%**
**0.01**	2.00	-0.31 – Inf	0.28*	-2.53 – (-0.15)
**0.1**	1.10	-0.99 – Inf	0.33*	-2.39 – 0.02
**1**	2.00	-0.31 – Inf	0.16*	-3.04 – (-0.74)
**10**	1.19	-0.93 – Inf	0.18*	-2.92 – (-0.62)
**100**	1.16	-0.94 – Inf	0.14*	-3.2 – (-0.91)

Table S3: Odds ratio (OR) values and their confidence intervals (CI) at 95% indicating the increased risk of mortality in zebrafish larvae exposed to PLA NPs according to the binomial logistic regression.

**mg/L**	**OR**	**CI 95%**
**0.01**	1.00	-2.62 – 2.62
**0.1**	2.07	-1.27 – 3.19
**1**	0.00	-Inf – 1.28
**10**	3.22	-0.65 – 3.58
**100**	2.07	-1.27 – 3.19

Table S4: Odds ratio (OR) values and their confidence intervals (CI) at 95% indicating the increased risk of malformation in zebrafish larvae exposed to PLA NPs according to the binomial logistic regression.

mg/L	OR	CI 95%
**0.01**	0.64	-2.15 – 1.12
**0.1**	1.80	-0.67 – 1.96
**1**	0.64	-2.15 – 1.12
**10**	1.80	-0.67 – 1.96
**100**	2.74	-0.19 – 2.34
